# Lubiprostone for the Treatment of Clozapine-Induced Constipation: A Case Series

**DOI:** 10.7759/cureus.25576

**Published:** 2022-06-01

**Authors:** Tyler J Torrico, Snehpreet Kaur, Manik Dayal, Pooja Eagala, David Weinstein

**Affiliations:** 1 Psychiatry, University of California Los Angeles Kern Medical Center, Bakersfield, USA; 2 Psychiatry, Ross University School of Medicine, Bakersfield, USA

**Keywords:** laxative, antipsychotic, gastric hypomotility, psychosis, schizophrenia

## Abstract

Clozapine-induced constipation is an increasingly recognized adverse reaction that frequently impairs optimal management of treatment-resistant schizophrenia. The Food and Drug Administration recently strengthened an existing warning for clozapine, citing constipation as an adverse effect that can progress to serious bowel complications. Evidence-based guidelines for laxatives in the management of clozapine-induced constipation remain scarce, and there is a general need for improved algorithms in the management of this common condition. Lubiprostone is a relatively new laxative that has labeled indications for opioid-induced constipation, irritable bowel syndrome with constipation, and chronic idiopathic constipation. This case series describes clinical pearls associated with four cases of treatment-resistant schizophrenia who underwent treatment of clozapine-induced constipation with lubiprostone. The findings of this case series suggest that there may be significant therapeutic potential in the utilization of lubiprostone for the management of clozapine-induced constipation with a low risk of adverse reactions. The study of lubiprostone benefit (i.e., without coadministration of other laxatives) continues to be of prominent interest in understanding its ability to manage clozapine-induced constipation.

## Introduction

Clozapine has been shown to be the most effective antipsychotic for treatment-resistant schizophrenia; however, it remains underutilized due to serious concerns over its adverse reaction profile [1]. The Food and Drug Administration recently strengthened an existing warning for clozapine, citing constipation as an adverse effect that can progress to serious bowel complications [2]. Shirazi et al. estimated the prevalence of clozapine-associated constipation at 31.2% of patients taking clozapine [3]. There is insufficient trial-based evidence to compare the effectiveness and safety of common laxatives for clozapine-induced constipation [4]. The risk of serious morbidity/mortality from clozapine-associated constipation is substantial, and there is a need for active monitoring of bowel function [5]. Due to the potential severity of clozapine-induced constipation, coadministration of laxatives is frequently encountered in clinical care [5,6]. Lubiprostone is currently approved by FDA for treatment of opioid-induced constipation, irritable bowel syndrome with constipation, and chronic idiopathic constipation. Lubiprostone has few adverse reactions, generally limited to diarrhea, nausea, and headache. Evidence for the use of lubiprostone in the treatment of clozapine-induced constipation is very limited [7-9] but demonstrates significant potential for its role in future laxative guidelines of clozapine-induced constipation. In this case series, we describe the clinical pearls of four case reports associated with the use of lubiprostone for the treatment of clozapine-induced constipation at our facility.

## Case presentation

Methods

Clozapine-induced constipation is a common problem that impairs optimal management of treatment-resistant schizophrenia in our community hospital located in Bakersfield, California. All patients were admitted to a behavioral health unit that had a no smoking policy. Between September 2021 and March 2022, our hospital treated 22 patients with clozapine, with four of these cases (18%) having severe clozapine-induced constipation, which was defined by constipation not being responsive to two common laxatives. For these cases, lubiprostone was utilized for the management of severe clozapine-induced constipation.

Case 1

**Figure 1:**
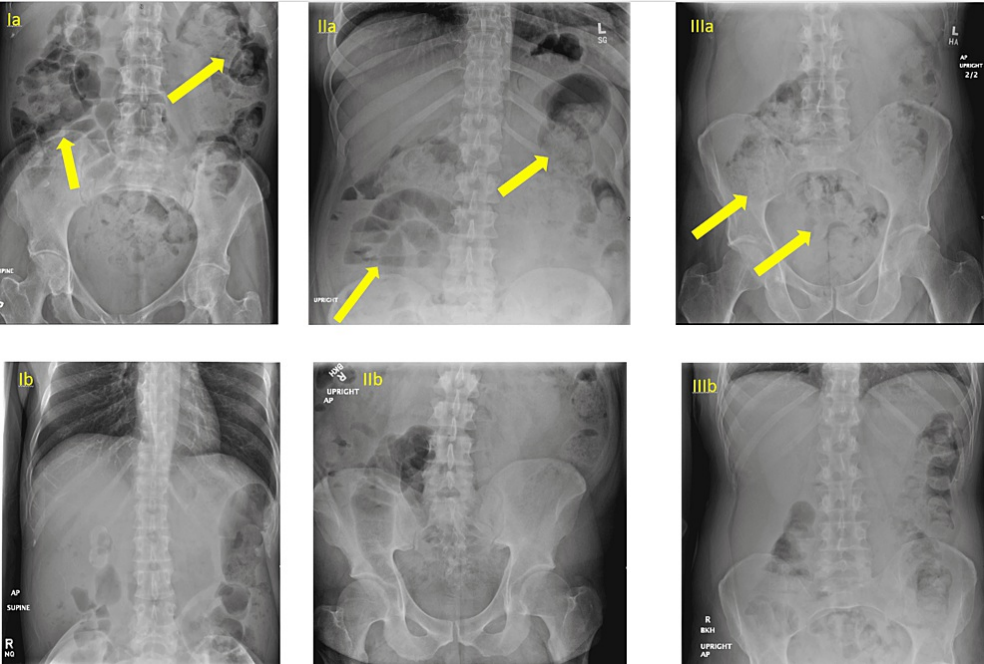
Abdominal X-rays of cases 1-3 Top row: Ia, IIa, and IIIa demonstrate abdominal X-rays of cases 1-3, respectively, before lubiprostone administration. Arrows indicate large areas of fecal stasis. Bottom row: Ib, IIb, and IIIb, demonstrate abdominal X-rays of cases 1-3, respectively, after lubiprostone administration.

A 61-year-old African American female with treatment-resistant schizophrenia was started on clozapine in the inpatient psychiatric unit. Clozapine was titrated over 11 days up to 200 mg daily when the patient reported no bowel movement for two days. An abdominal X-ray at that time revealed large colonic fecal matter (Figure 1-Ia) and was obtained due to the patient's low reliability as a historian due to disorganized speech secondary to her decompensated schizophrenia. The patient was started on docusate-senna 50-8.6 mg twice daily and lactulose 10 g three times daily and had one bowel movement.

Constipation returned as clozapine continued to be titrated to 350 mg daily on day 15. Lactulose was increased to 20 g three times per day, and daily fleet enema was attempted but without relief. By day 17, clozapine had been titrated to 400 mg and an abdominal X-ray (Figure 1-Ib) at this time raised concern for possible distal colonic obstruction, with general surgery consultation ultimately ruling out the colonic obstruction and recommending initiation of polyethylene glycol 17 g daily. On day 20 of titration with clozapine dose of 400 mg/day, lubiprostone 24 mcg twice daily was started. Clozapine was held the following day, and the patient had a small bowel movement. Clozapine was re-initiated at 300 mg daily, and the patient was able to have intermittent bowel movements on her discharge regimen of four laxatives (lubiprostone, polyethylene glycol, lactulose, docusate-senna). The patient had two separate episodes of bowel incontinence prior to discharge, with an abdominal X-ray in between these episodes demonstrating moderate colonic fecal content. 

Case 2

A 32-year-old Hispanic male with no significant medical history was admitted to the inpatient psychiatric unit and started on clozapine for treatment-resistant schizophrenia. Docusate-senna 50-8.6 mg twice daily and lactulose 10 g three times daily were prophylactically started on day 3 of clozapine titration. By day 16 of clozapine titration at 500 mg daily, the patient complained of worsening constipation with abdominal X-ray congruently showing moderately large residual fecal material with air-fluid levels (Figure 1-IIa). Docusate was titrated to 100 mg twice daily, and lactulose was increased to 20 g three times daily with no bowel movement over the next 24-hours. Lubiprostone 24 mcg twice daily and polyethylene glycol 17 g daily were started the following day. The abdominal X-ray on day 18 showed low to moderate colonic fecal volume, and the patient was able to have intermittent bowel movements for the duration of clozapine treatment (Figure 1-IIb). The patient was ultimately unable to continue treatment with clozapine due to the inability to engage with required blood count monitoring.

Case 3

A 62-year-old Caucasian male was admitted to the inpatient psychiatric unit with treatment-resistant schizophrenia, and he started on clozapine on day 4 of hospitalization while being cross-titrated from olanzapine 20 mg daily. Lactulose 20 g daily and docusate-senna 50-8.6 mg twice daily were prophylactically started on day 5. Clozapine was continued, but olanzapine was tapered off and discontinued on day 17. Congruently, lactulose was increased to 20 g twice daily due to worsened constipation, which adequately resolved symptoms. Psychosis remained uncontrolled, and clozapine was further titrated to 800 mg by day 50 of hospitalization. The patient, at this point, had not had a bowel movement for multiple days, and the abdominal X-ray showed a large fecal burden, which increased since prior X-rays were obtained for similar concerns (Figure 1-IIIa). Lubiprostone 24 mcg twice daily was started, and the patient showed improvement in constipation, with an abdominal X-ray on day 53 showing a reduction in fecal burden (Figure 1-IIIb). The patient tolerated this regimen for the remainder of their hospitalization.

Case 4

A 66-year-old Caucasian male with a medical history of hypertension, benign prostatic hyperplasia, and large renal mass (suspected renal cell carcinoma) was admitted to the inpatient psychiatric unit for decompensated treatment-resistant schizophrenia after self-discontinuing clozapine. A few months prior, the patient had previously been stable on clozapine 200mg daily with a simultaneous bowel regimen of docusate 100 mg daily. During this hospitalization, the patient's psychotic symptoms were severe, and he required stabilization on risperidone while undergoing clozapine titration (with the administration of docusate). The patient was quickly titrated on risperidone to 4 mg daily, which was transitioned to paliperidone palmitate 234 mg (the second dose of 156 mg was administered one week later). During this time, due to uncontrolled psychosis, the patient's clozapine dose was titrated past the previously well-tolerated 200 mg, which led to constipation becoming more prominent and requiring the addition of polyethylene glycol 17 g daily and lactulose 20 g three times daily. Due to severe persistent psychosis, further titration of clozapine was indicated, and the patient was initiated on lubiprostone 24 mcg twice daily to prevent further constipation. The patient was able to be titrated to a therapeutic dose of clozapine at 400 mg daily while having regular bowel movements on a combination of lubiprostone, docusate, and polyethylene glycol. No abdominal imaging was obtained for this patient's hospitalization.

## Discussion

Clozapine-induced constipation is a common and significant adverse reaction that impairs optimal management for treatment-refractory schizophrenia. Because clozapine is the most effective antipsychotic, decreasing the clozapine dose or switching to another antipsychotic when clozapine-induced constipation is severe may result in psychiatric decompensation rendering it an unfavorable but often medically necessary option. Despite this, evidence-based guidelines for laxatives in the management of clozapine-induced constipation remain scarce and are generally given subjectively at the individual clinicians' discretion [10,11].

This case series describes multiple clinical pearls to prescribing lubiprostone for the treatment of clozapine-induced constipation. Case 1 describes the relief of constipation with the addition of lubiprostone and decreased dose of clozapine. However, the patient did have incontinence in between episodes of constipation, potentially from the supratherapeutic effect of lubiprostone. Case 2 describes the relief of constipation with the addition of lubiprostone but had simultaneous initiation of polyethylene glycol, making it unclear if one agent or the combination of both was needed for the treatment of constipation. Case 3 and 4 describe the relief of constipation with the addition of lubiprostone to a regimen that included other laxatives. The described cases are suggestive of lubiprostone having a therapeutic benefit for the treatment of clozapine-induced constipation with a low risk of adverse reaction but are unable to assess its effectiveness as a monotherapy.

Patients with schizophrenia commonly have pre-existing risk factors for constipation, including dehydration, sedentary lifestyle, reduced fiber intake, and obesity [3,12]. The addition of clozapine further reduces gastrointestinal motility by strong blockade of muscarinic M3 receptors. This leads to colonic hypomotility by decreasing the uptake of calcium, inhibiting smooth muscle contraction, and leading to smooth muscle relaxation. The serotonergic antagonism of clozapine likely perpetuates this problem further and reduces nociception [3,13]. Instead of directly stimulating the enteric nervous system with stimulant laxatives, lubiprostone increases fluid secretion through the stimulation of chloride secretion into the intestinal lumen. This provides a bolus effect that softens stool and increases intestinal transit via stimulation of local receptors sensitive to stretch and distension, leading to improved contraction of peristaltic movements [14].

## Conclusions

The findings of this case series suggest that there may be significant therapeutic potential in the utilization of lubiprostone for the management of clozapine-induced constipation with a low risk of adverse reactions. The study of lubiprostone benefit (i.e., without coadministration of other laxatives) continues to be of prominent interest to understand its ability to manage clozapine-induced constipation alone. Limitations to this type of study in clinical practice include the wide accessibility of significantly cheaper and generally effective laxatives. Still, the potential for monotherapy treatment of clozapine-induced constipation is a worthwhile investigation as it may eliminate the need for polypharmacy treatment for an increasingly recognized complication of clozapine.
